# Contraction and expansion dynamics: deciphering genomic underpinnings of growth rate and pathogenicity in *Mycobacterium*

**DOI:** 10.3389/fmicb.2023.1292897

**Published:** 2023-11-23

**Authors:** Xiaoying Zhu, Qunfeng Lu, Yulei Li, Qinqin Long, Xinyu Zhang, Xidai Long, Demin Cao

**Affiliations:** ^1^Clinical Pathological Diagnosis & Research Center, The Affiliated Hospital of Youjiang Medical University for Nationalities, Baise, Guangxi, China; ^2^Medical College, Guangxi University, Nanning, Guangxi, China; ^3^Modern Industrial College of Biomedicine and Great Health, Youjiang Medical University for Nationalities, Baise, Guangxi, China; ^4^School of Medical Laboratory Sciences, Youjiang Medical University for Nationalities, Baise, Guangxi, China

**Keywords:** *Mycobacterium*, pathogenicity, growth rate, contraction/expansion, virulence factors, evolution

## Abstract

**Background:**

*Mycobacterium* bacteria, encompassing both slow growth (SGM) and rapid growth mycobacteria (RGM), along with true pathogenic (TP), opportunistic pathogenic (OP), and non-pathogenic (NP) types, exhibit diverse phenotypes. Yet, the genetic underpinnings of these variations remain elusive.

**Methods:**

Here, We conducted a comprehensive comparative genomics study involving 53 *Mycobacterium* species to unveil the genomic drivers behind growth rate and pathogenicity disparities.

**Results:**

Our core/pan-genome analysis highlighted 1,307 shared gene families, revealing an open pan-genome structure. A phylogenetic tree highlighted clear boundaries between SGM and RGM, as well as TP and other species. Gene family contraction emerged as the primary alteration associated with growth and pathogenicity transitions. Specifically, ABC transporters for amino acids and inorganic ions, along with quorum sensing genes, exhibited significant contractions in SGM species, potentially influencing their distinct traits. Conversely, TP strains displayed contraction in lipid and secondary metabolite biosynthesis and metabolism-related genes. Across the 53 species, we identified 26 core and 64 accessory virulence factors. Remarkably, TP and OP strains stood out for their expanded mycobactin biosynthesis and type VII secretion system gene families, pivotal for their pathogenicity.

**Conclusion:**

Our findings underscore the importance of gene family contraction in nucleic acids, ions, and substance metabolism for host adaptation, while emphasizing the significance of virulence gene family expansion, including type VII secretion systems and mycobactin biosynthesis, in driving mycobacterial pathogenicity.

## 1 Introduction

Within the phylum Actinomycetota, the genus *Mycobacterium* is diverse, comprising over 190 species. The cell walls of the *Mycobacterium* genus are characterized by a lipid-rich outer layer containing high concentrations of mycolic acid (Dulberger et al., 2020). Their unique composition necessitates the use of acid-fast staining to emphasize their resistance to acidic conditions, distinguishing them from other cell types (Pennington et al., 2021). However, bacteria within this genus exhibit highly distinctive phenotypic traits at various levels. Various types of mycobacteria are widely distributed across diverse environments, such as soil, water bodies, fish, amphibians, mammals, primates, and humans. Their classification into rapid grower mycobacteria (RGM) and slow grower mycobacteria (SGM) is determined by the growth of visible colonies on solid culture media within 7 days (Philley and Griffith, 2015). In addition to the well-known pathogens *M. tuberculosis* (causing tuberculosis) and *M. leprae* (causing leprosy), there are other species such as *M. marinum* and *M. shottsii* that induce diseases in fish and amphibians (Gauthier et al., 2021, 2022). Recent years have witnessed the emergence of opportunistic infections caused by diverse non-tuberculosis mycobacteria (NTM), introducing a new challenge in clinical therapy (Gopalaswamy et al., 2020; Ratnatunga et al., 2020). RGM are predominantly environmental saprophytes, whereas all pathogenic bacteria fall within the slow growth mycobacteria category. However, opportunistic pathogens are distributed across all types.

Distinct genetic factors underpin the pathogenicity, growth rate, and remarkable adaptability of bacteria to diverse habitats. Conducting comparative genomic analysis is an effective approach to comprehensively decipher the genotype–phenotype relationship in bacteria and understand the interplay between species’ genomic evolution and their environment. Our previous study revealed that the capacity to utilize diverse carbohydrates, enabling *Listeria monocytogenes* and *L. ivanovii* to effectively adapt to and exploit various carbohydrate sources in their environment, represents a crucial genetic trait contributing to their pathogenicity (Lu et al., 2022). By conducting a genomic comparison between SGM and RGM, Bachmann et al. discovered that the absence of *livFGMH*, *shaAC-G*, and ABC transporter operons, which are associated with amino acid and ion transport in SGM, may contribute to alterations in their growth rate (Bachmann et al., 2019). Recent comparative genomic studies have highlighted the pivotal role of bacterial genome expansions or contractions in driving modifications in their biological capabilities (Baroncelli et al., 2016). Notably, the *pe* and *ppe* gene families, with the majority of their members secreted through the Type-VII secretion system, undergo significant expansion in pathogenic *Mycobacterium* species (Cole et al., 1998). These gene families play vital roles in maintaining iron homeostasis. For example, PE5-PPE4 is implicated in mycobactin-mediated iron acquisition, whereas PPE36 and PPE37 are linked to heme-iron acquisition, among other functions (Tufariello et al., 2016; Tullius et al., 2019). These expansions within the gene families are likely to contribute to the virulence and pathogenicity of *Mycobacterium*. Nonetheless, our current comprehension of how gene family expansion and contraction influence the evolution of pathogenicity and environmental adaptability in mycobacteria remains incomplete. Further exploration at a broader genomic level is warranted.

Recently, a considerable number of complete genomes from various *Mycobacterium* species have been sequenced. This wealth of genomic data provides a valuable opportunity to obtain comprehensive insights into the genetic foundation and evolutionary attributes underlying the intricate diversity of *Mycobacterium* phenotypes. Such insights hold potential implications for the prevention and treatment of *Mycobacterium* infections. In this study, we conducted a comparative genomic study using representing complete genomes of 53 *Mycobacterium* species. In the present study, we performed a comparative genomic analysis utilizing complete genomes representing 53 distinct *Mycobacterium* species. These species were stratified into two groups according to their growth rates: SGM and RGM. Furthermore, an additional categorization was established based on their pathogenicity, dividing them into three groups: Totally Pathogens (TP), Opportunity Pathogens (OP), and Non-Pathogens (NP). Subsequently, core/pan-genome analysis and functional annotation were conducted, yielding insights into the genomic structure and functional makeup of bacteria within this genus. Next, a phylogenetic tree was constructed utilizing single-copy core genes, and the gene families undergoing contraction/expansion for each node were estimated. Additional analysis, considering gene function and protein interactions, elucidates the role of gene expansion/contraction events in influencing pathogenicity and environmental adaptability. Lastly, we investigated the distribution and expansion/contraction of virulence factors within each mycobacterial species. Our findings uncover the pivotal role of gene family contraction in the transition from RGM to SGM, as well as in the acquisition of pathogenicity.

## 2 Materials and methods

### 2.1 Data retrieval and genome management

The complete-genome sequences of 53 *Mycobacterium* species were obtained from the genome database at NCBI,^1^ with data retrieved until May 5, 2023. To ensure analytical consistency, we re-annotated the structure of each genome using the same pipeline. Specifically, the open reading frames of each genome were identified using Prodigal V2.6.3 (Hyatt et al., 2010). The identification of tRNAs was conducted with tRNAscan-SE 1.3.1, while rRNAs were identified using rnammer 1.2 (Lagesen et al., 2007). Key genome attributes, including genome size, GC-content, number of coding sequences (CDS), growth type, pathogenicity, and other pertinent features, were documented and are outlined in Table 1.

**Table 1:** The genome features of 53 representing *Mycobacterium* species included in this study.

Genus	Strain ID	Accession number	Genome size (bp)	GC content (%)	# of scaffold	# of plasmid	CDS	rRNA	tRNA	Growth type^a^	Pathogenicity^b^
*M. tuberculosis*	H37Rv	GCA 000195955.2	4,411,532	65.61	1	0	3,981	3	45	SGM	TP
*M. avium*	104	GCA 000014985.1	5,475,491	68.99	1	0	5,175	3	46	SGM	OP
*M. fortuitum*	JCM 6387	GCA 022179545.1	6,406,072	66.19	1	0	6,134	6	55	RGM	OP
*M. intracellulare*	ATCC 13950	GCA 000277125.1	5,402,402	68.1	1	0	5,003	3	47	SGM	OP
*M. kansasii*	ATCC 12478	GCA 000157895.2	6,577,228	66.23	1	1	5,723	3	46	SGM	TP
*M. leprae*	TN	GCA 000195855.1	3,268,203	57.8	1	0	3,896	3	45	SGM	TP
*M. chelonae*	CCUG 47445	GCA 001632805.1	5,029,817	63.92	1	0	4,827	3	47	RGM	OP
*M. marinum*	E11	GCA 000723425.2	6,450,522	65.74	1	1	5,328	6	48	SGM	TP
*M. ulcerans*	Agy99	GCA 000013925.2	5,805,761	65.39	1	1	5,397	3	45	SGM	TP
*M. haemophilum*	DSM 44634 ATCC 29548	GCA 000340435.3	4,235,765	63.95	1	0	3,897	3	45	RGM	OP
*M. abscessus*	GZ002	GCA 004028015.1	5,082,434	64.14	1	1	4,918	3	47	RGM	OP
*M. gallinarum*	JCM 6399	GCA 010726765.1	6,301,681	65.61	1	1	6,152	6	46	RGM	NP
*M. branderi*	JCM 12687	GCA 010728725.1	5,979,623	66.49	1	1	5,742	6	46	SGM	OP
*M. conspicuum*	JCM 14738	GCA 010730195.1	6,237,139	67.39	1	0	5,652	3	48	SGM	OP
*M. heidelbergense*	JCM 14842	GCA 010730745.1	5,050,576	67.94	1	0	4,583	3	45	SGM	OP
*M. canettii*	CIPT 140010059	GCA 000253375.1	4,482,059	65.62	1	0	3,960	3	45	SGM	TP
*M. heckeshornense*	JMUB5695	GCA 016861545.1	4,865,109	65.9	1	0	4,557	6	46	SGM	OP
*M. frederiksbergense*	LB 501 T	GCA 012223425.1	6,713,618	67.07	1	3	6,521	6	46	RGM	NP
*M. kubicae*	NJH MKUB1	GCA 014263315.1	5,463,479	66.28	1	2	5,075	3	49	SGM	OP
*M. shottsii*	JCM 12657	GCA 010728525.1	5,973,149	65.53	1	0	5,625	3	47	SGM	TP
*M. lentiflavum*	ATCC 51985	GCA 022374895.2	6,163,560	65.94	1	3	5,650	3	47	SGM	OP
*M. lacus*	JCM 15657	GCA 010731535.1	5,092,988	66.95	1	0	4,605	3	45	SGM	OP
*M. parmense*	JCM 14742	GCA 010730575.1	5,952,912	68.39	1	0	5,435	3	49	SGM	NP
*M. saskatchewanense*	JCM 13016	GCA 010729105.1	6,008,916	68.28	1	0	5,574	3	48	SGM	OP
*M. liflandii*	ASM001	GCA 022354805.1	6,167,296	65.57	1	0	5,641	3	47	SGM	TP
*M. pseudoshottsii*	JCM 15466	GCA 003584745.1	6,061,597	65.64	1	0	5,430	3	47	SGM	TP
*M. florentinum*	JCM 14740	GCA 010730355.1	6,219,859	66.38	1	0	5,805	3	47	SGM	OP
*M. colombiense*	CECT 3035	GCA 002105755.1	5,581,643	68.09	1	0	5,300	3	47	SGM	OP
*M. seoulense*	JCM 16018	GCA 010731595.1	5,531,300	68.29	1	0	5,087	3	48	SGM	NP
*M. shinjukuense*	JCM 14233	GCA 010730055.1	4,504,020	67.79	1	0	3,984	3	45	SGM	OP
*M. salmoniphilum*	DSM 43276	GCA 004924335.1	4,776,697	64.29	1	0	4,572	3	56	RGM	NP
*M. noviomagense*	JCM 16367	GCA 010731635.1	4,779,798	65.76	1	0	4,504	6	45	SGM	OP
*M. stomatepiae*	JCM 17783	GCA 010731715.1	6,210,822	65.96	1	0	5,981	3	48	SGM	OP
*M. lepromatosis*	FJ924	GCA 000975265.2	3,271,694	57.89	1	0	3,806	3	46	SGM	TP
*M. dioxanotrophicus*	PH-06	GCA 002157835.1	8,080,416	66.46	1	4	7,725	9	83	RGM	NP
*M. riyadhense*	NTM	GCA 016864455.1	6,772,223	65.1	1	0	5,998	3	48	SGM	TP
*M. mantenii*	JCM 18113	GCA 010731775.1	6,185,541	66.88	1	0	5,674	3	48	SGM	OP
*M. paraterrae*	DSM 45127	GCA 022430545.2	5,522,624	65.55	1	0	5,193	6	48	SGM	NP
*M. paraseoulense*	JCM 16952	GCA 010731655.1	6,085,955	67.9	1	0	5,641	3	46	SGM	NP
*M. marseillense*	FLAC0026	GCA 002285715.1	5,285,642	67.84	1	1	4,842	3	47	SGM	OP
*M. shigaense*	JCM 32072	GCA 002356315.1	5,232,660	67.26	1	0	4,871	3	47	SGM	OP
*M. litorale*	NIIDNTM18	GCA 014218295.1	5,634,149	68.88	1	0	5,407	6	47	RGM	NP
*M. spongiae*	FSD4b-SM	GCA 018278905.1	5,581,157	65.56	1	0	4,472	3	45	SGM	NP
*M. paraintracellulare*	M011	GCA 016756055.1	5,357,612	68.12	1	1	4,968	3	46	SGM	NP
*M. paragordonae*	49,061	GCA 003614435.1	7,224,251	66.89	1	4	6,411	3	47	SGM	OP
*M. stephanolepidis*	NJB0901	GCA 002356335.1	4,994,485	63.95	1	0	4,916	3	49	RGM	NP
*M. grossiae*	DSM 104744	GCA 008329645.1	5,681,602	70.45	1	1	5,382	6	46	RGM	NP
*M. saopaulense*	EPM10906	GCA 001456355.1	4,649,175	64.8	1	0	4,488	3	49	RGM	NP
*M. vicinigordonae*	24	GCA 013466425.1	6,266,765	65.35	1	0	5,577	3	46	SGM	NP
*M. basiliense*	901,379	GCA 900292015.1	5,607,630	65.02	1	0	4,696	3	48	SGM	OP
*M. novum*	JCM 6391	GCA 010726505.1	4,458,926	68.58	1	0	4,161	9	46	SGM	NP
*M. ostraviense*	FDAARGOS 1613	GCA 021183725.1	6,127,734	66.27	1	0	5,536	3	46	SGM	NP
*M. senriense*	TY59	GCA 019668465.1	5,831,451	67.08	3	0	5,339	3	47	SGM	NP

a SGM, slow growth mycobacteria; RGM, rapid growth mycobacteria.

b TP, totally pathogen; OP, opportunity pathogen; NP, not pathogen.

### 2.2 Core- and pan-genome analysis

The homologous genes were identified using OrthoFinder version 2.5.4, employing an all-*vs*-all BLASTp search based on the proteome of each genome (Emms and Kelly, 2019). Subsequently, we computed the proportion of homologous genes both between species and within species using the following formulas:


(1) $$\eta =\frac{2N_{s}}{\left(N_{a}+N_{b}\right)}\times 100\%\text{,}$$

where η is the proportion of homologous genes, *N_s_* is the number of homologous genes, *N_a_* is the number of proteins of one genome, and *N_b_* is the number of proteins of another genome. The results were visualized with a heatmap by r-ggplot2.

Pan-genome size can be determinated by a prediction using Heap’s law, which is formulated as:


(2) $$y=A_{1}x^{-B1}+C_{1}\text{,}$$

where y is the pan-genome size, x is the number of genomes used, and *A1, B1, C1* are the fitting parameters.

Core-genome size can be determinated by a prediction using power law, which is formulated as:


(3) $$y=A_{2}x^{B2}+C_{2}\text{,}$$

where y is the core-genome size, x is the number of genomes used, and *A2, B2, C2* are the fitting parameters. The fitted curve of core and pan-genome were displayed using R version 4.2.1.

### 2.3 Phylogenetic analysis

Multiple amino acid sequence alignments of 507 single-copy genes from the 53 *Mycobacterium* species were generated using MAFFT v7.490 (Katoh and Standley, 2013). Highly divergent sites were subsequently removed using Clipkit v1.3.0 (Steenwyk et al., 2020). Subsequently, a maximum likelihood (ML) phylogenetic tree was constructed using the best-fit substitution model JTT + F + R7, as determined by ModelFinder (Kalyaanamoorthy et al., 2017), implemented in IQ-TREE v1.6.12 (Nguyen et al., 2015). Divergence times between species were estimated using the MCMC tree program in paml-v4.10.6 (Yang, 2007). To deduce alterations in gene family size, we utilized the program CAFE5-5.1.0 (Mendes et al., 2020). The topology of the ML tree was visualized using ggtree v3.6.2 (Yu et al., 2016). The growth type and pathogenicity of each species were displayed on the right side of the tree, while gene family contraction/expansion events were depicted using pie charts on the corresponding nodes of the tree.

### 2.4 Functional annotation of core- and accessory-genome

To annotate the genes within the core and accessory genome using Clusters of Orthologous Groups (COG), a whole-genome BLASTp search was performed against both the NCBI COG database (v20) and EggNOG v5.0 (Huerta-Cepas et al., 2019), employing an E-value cutoff of 1e-5. Subsequently, the results were integrated using a Python script.

### 2.5 Annotation of virulence factor and antibiotic resistance genes

The proteins encoded by genes from the 53 mycobacterial species underwent a BLASTp search against the virulence factor database (VFDB) to identify potential virulence-related genes (Liu et al., 2022). For the identification of antibiotic resistance genes within the genomes of the 53 *Mycobacterium* species, the genes were aligned against the comprehensive antibiotic resistance database (CARD) (Alcock et al., 2019). The BLASTp search was conducted with parameters set to 90% identity, 90% coverage, and an E-value cutoff of 1e-5. The outcomes were visualized using ComplexHeatmap v2.14.0 (Gu, 2022).

### 2.6 Analysis of protein–protein interactions

Protein–protein interaction networks for the sets of genes were constructed using the Search Tool for the Retrieval of Interacting Genes/Proteins (STRING v12.0, https://string-db.org/, accessed on 9 July, 2023) (Szklarczyk et al., 2021). Additionally, Cytoscape 3.9.1 was employed for visualizing these networks (Shannon et al., 2003).

### 2.7 Data availability statement

All data analyzed and generated throughout this study have been comprehensively integrated into both the manuscript and Supplementary material.

## 3 Results and discussion

### 3.1 Genome statistics and general features

The *Mycobacterium* genus exhibits significant species diversity and notable variation in phenotypic characteristics. The List of Prokaryotic Names with Standing in Nomenclature database (LPSN, https://www.bacterio.net/genus/mycobacterium, accessed until June 27, 2023) documents 195 taxa of *Mycobacterium*. To comprehensively explore the genomic diversity of the *Mycobacterium* genus, this study meticulously selected and analyzed complete-level genomes from 53 *Mycobacterium* species (Table 1). The genomes within this genus featured elevated GC content, with an average of 63.17%. The genome of *L. leprae* had the lowest GC content (57.8%), while *M. grossiae* had the highest GC content (70.45%) among them. The presence of plasmids was infrequent within *Mycobacterium* genomes, with only fifteen out of fifty-three species detected with plasmids. All strains were classified into SGM and RGM based on their growth rates. Out of these *Mycobacterium* species, 41 were SGM and 12 were RGM. Furthermore, the 53 species were categorized into TP, OP and NP based on their virulence. Among these, eleven species were categorized as TP, encompassing *M. tuberculosis, M. canettii, M. kansasii, M. leprae, M. leproma, M. ulcerans*—organisms that infect humans or other mammals, and *M. marinum, M. shottsii, M. liflandii, M. pseudoshottsii*, which infect fish or amphibians (Gauthier et al., 2021; Bartlett et al., 2022). A total of twenty-four species were classified as OP, implicated in causing lung or other infections in immunocompromised patients. The remaining eighty species were NP species, which rarely cause infection disease. In the natural context, pathogenic bacteria or specific parasitic bacteria typically exhibit smaller genomes compared to free-living species (Ochman and Davalos, 2006). This principle can also be extended to the analysis of *Mycobacterium* genomes.

### 3.2 Composition and functional characteristics of the pan-genome in 53 *Mycobacterium* species

**Figure 1:**
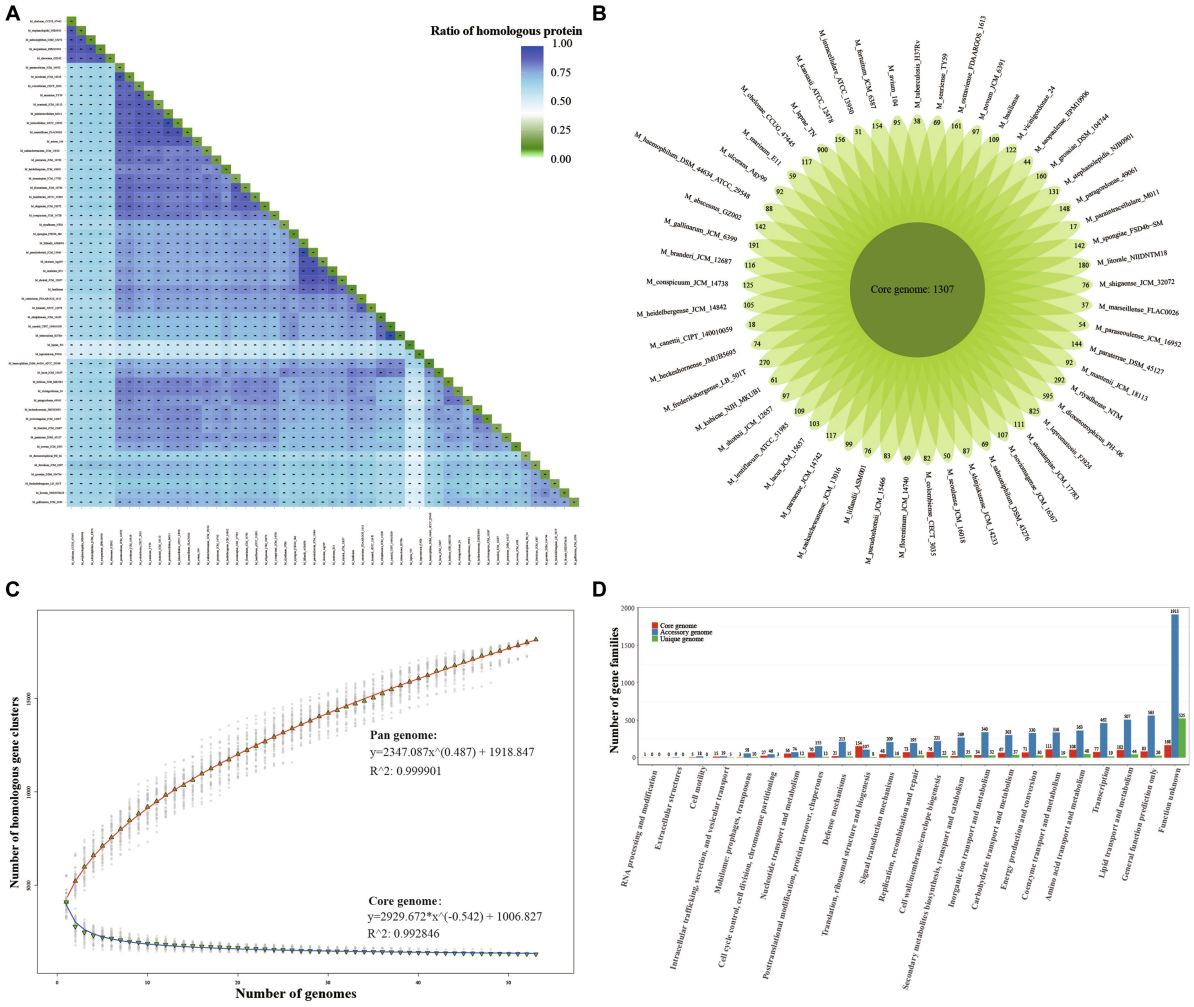
Core/Pan-genome structure and gene function distribution. **(A)** Percentage of homologous gene families for each pair of *Mycobacterium* species. The corresponding tiles display the percentage and number of homologous gene families, as well as the mean number of gene families, for each species pair. **(B)** Core genome size and unique gene sizes for each *Mycobacterium* species. **(C)** Cumulative size curve for core (blue) and pan-genome (red). The blue curve represents the cumulative sizes of the core genome, while the red curve depicts the cumulative sizes of the pan-genome. **(D)** COG categories of core, accessory, and unique genomes for *Mycobacterium*. The distribution of gene function categories within the core, accessory, and unique genomes of *Mycobacterium*.

To assess the genetic diversity among *Mycobacterium* species, we conducted an analysis of homologous genes for each pair of species (Figure 1A, Supplementary Table S1). *M. tuberculosis* and *M. canettii* exhibited the highest proportion of homologous genes, reaching 97.27%. This observation signifies a close relationship between these two species (Supply et al., 2013). It is evident that *M. chelonae, M. stephanolepidis, M. salmoniphilum, M. saopaulense, M. abscessus* exhibit noteworthy distinctions from other species, yet they share a closer relationship among themselves. In addition, the smallest genomes, *M. leprae* and *M. lepromatosis,* exhibited the lowest proportion of homologous genes shared with other species. Regarding homology within the genome, *M. dioxanotrophicus* displayed 19.52% homologous genes, whereas *M. haemophilum* had 9.16% ones. Notably, pathogens such as *M. canettii* (9.61%), *M. tuberculosis* (9.62%), and *M. leprae* (9.33%) exhibited a low proportion of homologous genes within their genomes. This trend might be attributed to their prolonged adaptive evolution within parasitic environments (Ochman and Davalos, 2006).

From the outcomes of the analysis on homologous gene families, we identified a total of 18,139 gene families. Among these, 1,307 (7.21%) were shared across all 53 *Mycobacterium* species, 9,266 (51.08%) constituted the accessory genome, being shared by at least two but not all species, and the remaining 7,566 (41.71%) were characterized as singleton genes (Figure 1B). In essence, the core genes are accountable for the fundamental biological processes of a species and their key phenotypic traits. On the contrary, accessory and unique genes could potentially encode supplementary metabolic pathways that are not essential for survival but confer selective advantages (Medini et al., 2005; Tettelin et al., 2008). The prevalence of non-essential genomic content could potentially serve as the genetic foundation for *Mycobacterium* species to adapt to intricate environments and manifest diverse phenotypes. Interestingly, despite the small genomes of *M. leprae* and *M. lepromatosis*, they contained 900 and 825 singleton genes, respectively. This observation indicates distinct profiles in their genome composition. By means of curve fitting analysis, we derived the best-fitting function for core-genome size as y = 2929.672*χ^(−0.542)^ + 1006.827, and for pan-genome size as y = 2347.087*χ^(0.487)^ + 1918.847 (Figure 1C). Evidently, the core genome size reached a plateau after encompassing 30 species, whereas the pan-genome size exhibited rapid expansion as the number of species increased. This observation indicates that the *Mycobacterium* genus features an open pan-genome, highlighting a significant level of interspecific genetic diversity within *Mycobacterium*.

To explore whether the core, accessory, and unique genomes within the pan-genome exhibit distinct functional characteristics, we conducted an integrated functional annotation analysis using COG and EggNOG databases. As depicted in Figure 1D, the core-genome exhibited the two most enriched COG functional categories: “Translation, ribosomal structure, and biogenesis” (11.79%, 154/1307) and “Coenzyme transport and metabolism” (8.50%, 111/1307). In contrast, the proportions of these three categories within the accessory and unique genomes were 1.15, 3.62, and 0.11%, 0.25%, respectively. Regarding the accessory genome, the most enriched COG categories were “Lipid transport and metabolism” (5.47%) and “Transcription” (4.99%). This observation aligns with the anticipated notion that the core genome primarily encompasses housekeeping functions, as opposed to the accessory and unique genes. Furthermore, a notable prominence of “Mobilome: prophages, transposons,” “Defense mechanisms,” and “Signal transduction mechanisms” was evident within the accessory genome. These elements potentially contribute to their diverse niche adaptation and pathogenicity. However, it is noteworthy that 53.91% of accessory gene families and 94.81% of unique gene families lacked specific COG functional categorizations. This observation underscores the limited comprehension of gene function within *Mycobacterium* species, warranting further investigations in the future.

### 3.3 Expansion and contraction of gene families in *Mycobacterium* species

**Figure 2:**
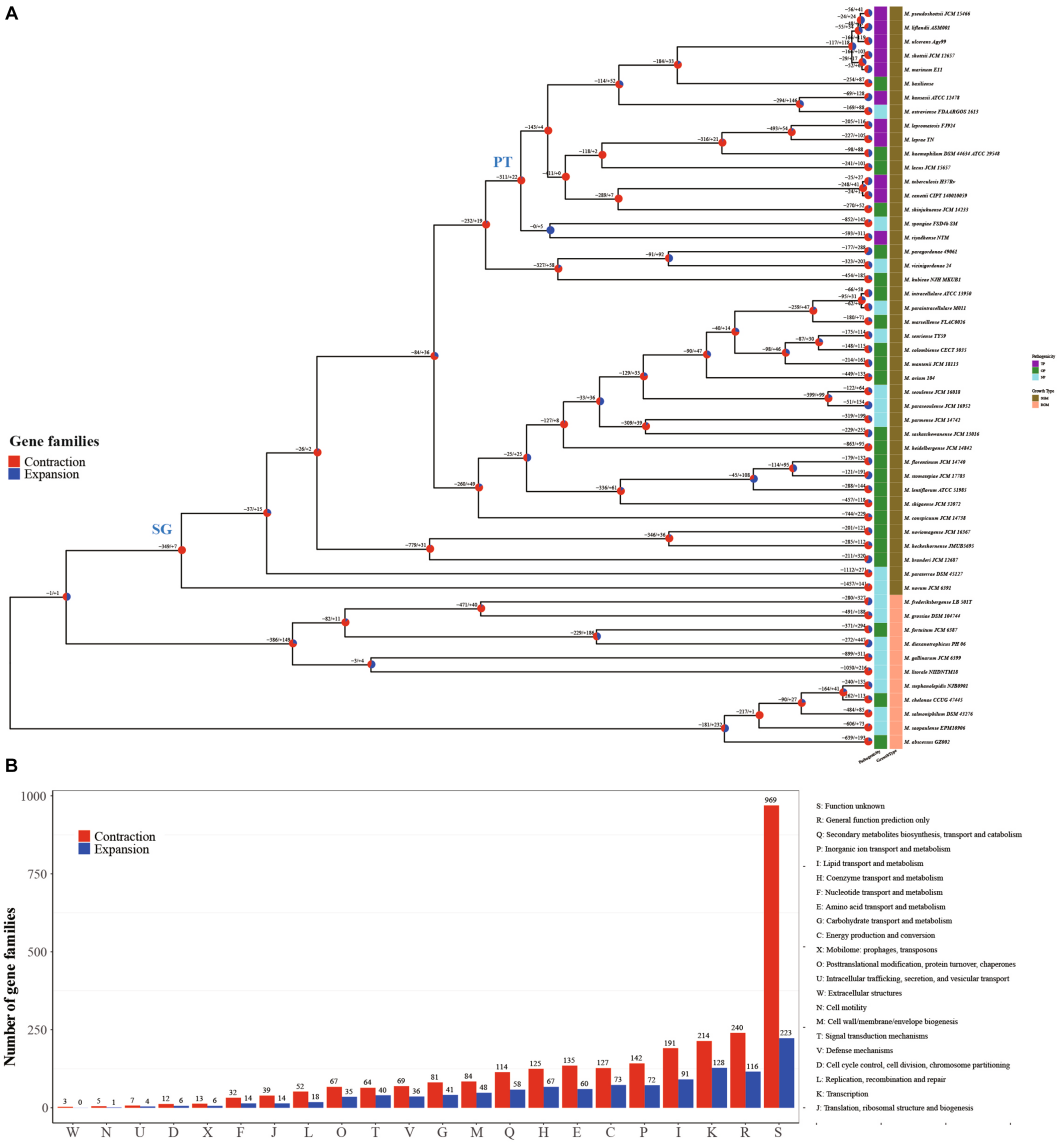
Structure and function distribution of gene family expansion and contraction in each *Mycobacterium* lineage. **(A)** Maximum-Likelihood tree constructed using concatenated amino acid sequences of 719 single-copy core genes. Each node on the tree is represented by a pie chart indicating the percentage of gene family contraction (red) and expansion (blue). The heatmap on the right of each species node depicts pathogenicity and growth type information. **(B)** Distribution of COG categories for contraction and expansion gene families of all inner nodes.

Remarkably, *Mycobacterium* species exhibit close genetic relationships while manifesting notable differences in phenotypes, including growth rate and pathogenicity (Pepperell, 2022). For a more comprehensive grasp of the evolutionary patterns among *Mycobacterium* species, we constructed a phylogenetic tree utilizing 709 single-copy core genes from the genomes of 53 *Mycobacterium* species. This was accomplished through the maximum likelihood method employing the JTT + F + R7 model (Figure 2A). The constructed tree substantiated the close relationship among all the fully pathogenic species, clustering them within the pathogenic mycobacteria (PT) node of the tree. This group encompassed *M. tuberculosis, M. leprae, M. marinum, M. ulcerans, M. canettii, M. shottsii* and others. *M. canettii* was identified as the species closest to *M. tuberculosis*. Notably, *M. ulcerans*, responsible for Buruli ulcer, formed a cluster alongside *M. shottsii, M. liflandii, M. pseudoshottsii*, and *M. marinum*—pathogens affecting fish and amphibians (Fremont-Rahl et al., 2011; Gauthier et al., 2022). These findings align with prior phylogenetic research grounded in average nucleotide identity (ANI) (Tortoli et al., 2017). A distinct demarcation exists between RGM and SGM species. All SGM species were positioned within the branches of the slow growth mycobacteria (SG) node on the tree. The *M. abscessus* complex constituted the most ancestral cluster of RGM, whereas *M. novum* emerged as the most ancestral species among SGM. The SGM group encompassed all TP species and a majority of the opportunity pathogenic species.

Gene family expansion and contraction constitute critical genetic factors influencing the habitat range and pathogenicity of bacteria (Baroncelli et al., 2016; Zhong et al., 2022). In this study, we reconstructed the genome-wide history of gene family contraction and expansion events across 53 *Mycobacterium* species. This reconstruction was conducted using the phylogenetic tree and encompassed all gene families within the pan-genome (Figure 2A). Our analysis revealed the participation of a total of 3,353 gene families (18.49%, 3,353/18,139) in contraction and expansion events. In the transition from RGM to SGM species, a majority of evolutionary events (77.78%) occurring at inner nodes involved gene family contractions. Specifically, there were 2,841 expansion events, encompassing 1,047 non-redundant gene families, alongside 9,947 contraction events, involving 2,580 non-redundant gene families, observed across the inner nodes. This outcome underscores the pivotal role of gene family contraction in the differentiation and formation of *Mycobacterium* species. Functional annotation using COG categories demonstrated the enrichment of “Transcription” and “Lipid transport and metabolism” within the gene families undergoing contraction and expansion events at inner nodes (Figure 2B). The regulation of gene expression stands as a pivotal mechanism for bacteria to adapt to intricate environments. It is widely recognized that the characteristic high lipid content, featuring diverse structures and biological activity, is a hallmark of *Mycobacterium* species (Lanéelle et al., 2021), contributing significantly to their survival and pathogenesis (Mallick et al., 2021). In contrast to inner nodes, the quantity of each COG category for contraction and expansion gene families within terminal branches was nearly uniform (Supplementary Figure S1). This variation in distribution patterns suggests that the gene families’ contraction events play a significant role in the evolution and formation of *Mycobacterium* species.

### 3.4 Gene families associated with growth and pathogenicity traits in *Mycobacterium*

The evolutionary tree of *Mycobacterium* species distinctly separates RGM and SGM species, alongside TP, OP, and NP categories (Figure 2A). Crucially, the SG node and PT node served as pivotal points for the emergence of phenotypic alterations in growth rate and pathogenicity, respectively. From a logical standpoint, the contraction and expansion gene families associated with these nodes are intricately connected to the growth rate and pathogenicity characteristics of mycobacteria. In light of this, we meticulously conducted a comprehensive functional analysis of the contraction and expansion gene families linked to the SG and PT nodes. Virtually all of these events materialized as gene family contractions. To be specific, the counts of contraction and expansion gene families for the SG node were 349/7, whereas for the PT node, they stood at 311/22.

**Figure 3:**
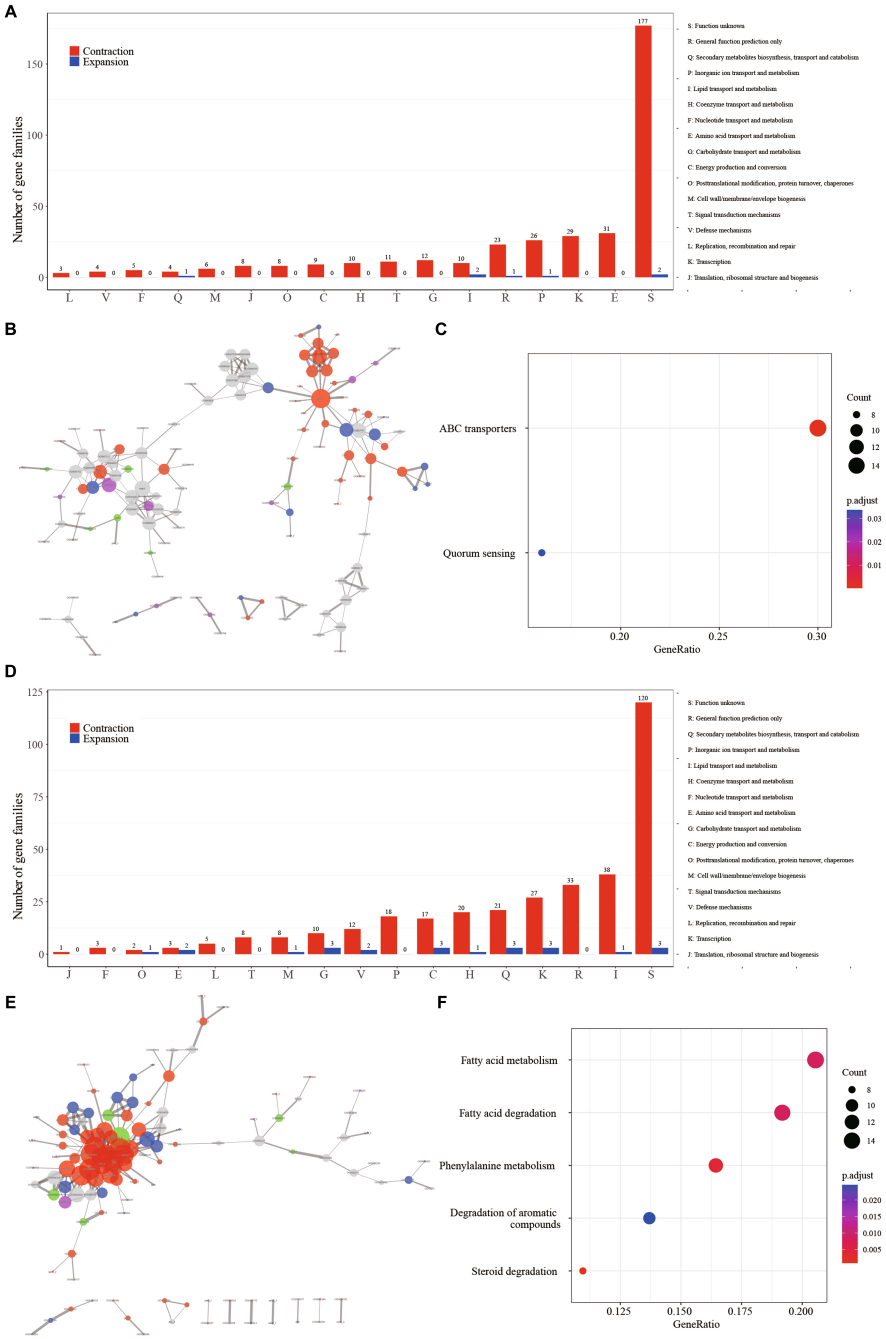
The major events in the context of growth speed type alteration and pathogenicity transition are characterized by the contraction of gene families. **(A)** Distribution of COG categories among contraction (depicted in red) and expansion (depicted in blue) gene families for GT node. **(B)** Protein–protein interaction network of contraction gene families for GT node. Network edges illustrate protein–protein interactions, with line thickness indicating the confidence level of the relationships between nodes. Node size corresponds to the node degree. Nodes are color-coded to represent diverse protein function categories: red for basic substance transport and metabolism, purple for genetic information processing (including replication, transcription, and translation), blue for cellular processes (including cell wall/membrane/envelope biogenesis and cell motility), green for bacteria-environment interaction (including signal transduction, extracellular structures, and defense mechanisms), and grey for proteins lacking meaningful function annotation. **(C)** Enriched KEGG metabolism network of contraction gene families for GT node. **(D)** Distribution of COG categories among contraction (depicted in red) and expansion (depicted in blue) gene families for PT node. **(E)** Protein–protein interaction (PPI) network of contraction gene families for PT node. **(F)** Enriched KEGG metabolism network of contraction gene families for PT node.

Regarding the SG node, the most prominently enriched COG categories among the contraction gene families included “E: Amino acid transport and metabolism” (8.82%, 31/349), “K: Transcription” (8.31%, 29/349), and “P: Inorganic ion transport and metabolism” (7.45%, 26/349). However, a notable proportion of the gene families (50.28%, 179/356) lacked definitive annotation results (Figure 3A, Supplementary Table S2). The protein–protein interaction (PPI) network revealed the aggregation of these gene families into two distinct clusters (Figure 3B). A majority of these gene families were associated with fundamental bacterial substance transport and metabolic processes. Gene ontology (GO) enrichment analysis highlighted the substantial enrichment of gene families in ATP binding cassette (ABC) transporters and Quorum sensing (QS) (Figure 3C). Fifty gene families were implicated in ABC transporters, which play a pivotal role in bacterial physiology by facilitating the transport of diverse substrates impacting nutrition, pathogenesis, and antibiotic resistance (Orelle et al., 2019; Cassio Barreto De Oliveira and Balan, 2020). For instance, the ABC transporter encoded by the gene Rv1819c can transport unrelated hydrophilic compounds such as bleomycin and cobalamin, which are linked to the pathogenesis of tuberculosis (Rempel et al., 2020). The contraction of ABC transporter-related gene families in SGM could potentially diminish the efficiency of substance transport and metabolism, thereby contributing to their slow growth phenotypes. Facilitates biofilm formation, aiding bacterial survival in intricate environments (Sharma et al., 2014). Numerous *Mycobacterium* species, including *M. abscessus*, *M. avium*, *M. marinum,* and *M. fortuitum*, have demonstrated the ability to form biofilms across diverse conditions (Bardouniotis et al., 2003; Johansen et al., 2009). The contraction of gene families related to QS could signify an adaptive evolutionary response to a slow-growth state.

In contrast to the SG node, where gene contraction was predominantly associated with amino acid transport and metabolism, the PT node exhibited a distinct pattern. Here, gene families related to “I: Lipid transport and metabolism” (12.22%, 38/311), “R: Transcription (8.68%, 27/311),” and “Q: Secondary metabolites biosynthesis, transport and catabolism (6.75%, 21/311)” were prominently enriched, suggesting a strong link between these processes and pathogenicity (Figure 3D, Supplementary Table S3). The observation of these gene families clustering together in the protein–protein interaction network (Figure 3E) underscores their functional interconnectedness. This cluster primarily encompasses critical functions related to fundamental substance transport, metabolism, and cellular processes. Furthermore, the gene ontology (GO) enrichment analysis highlighted significant enrichment of five metabolic pathways in this gene cluster: “Fatty acid metabolism,” “Fatty acid degradation,” “Phenylalanine metabolism,” “Degradation of aromatic compounds,” and “Steroid degradation” (Figure 3F). Furthermore, the pivotal regulator FdmR in the pathogen *M. marinum* governs the abundance and chain length of virulence-associated lipids and mycolates, critically contributing to the maintenance of cell envelope impermeability. Notably, the role of fatty acid metabolism emerged as pivotal. In particular, the regulator *FdmR* in the pathogen *M. marinum* was highlighted for its control over the abundance and chain length of virulence-associated lipids and mycolates. This regulatory function is crucial for maintaining the impermeability of the cell envelope, a vital aspect of pathogenicity (Dong et al., 2021). Moreover, Yang et al. (2021) revealed that the hypoxia-induced mycobacterial protein, fatty-acid degradation A (FadA), plays a role in suppressing host immunity by modulating host fatty acid metabolism. This underscores the significance of fatty acid metabolism in mycobacteria’s adaptation to the host’s internal environment and in generating pathogenicity. Previous studies have demonstrated that members of the genus *Mycobacterium* are highly proficient in degrading aromatic compounds, which are prevalent environmental contaminants posing potential risks to human health (Kweon et al., 2015; Hennessee and Li, 2016). Interestingly, it’s worth noting that mycobacteria, a genus known for its proficiency in degrading aromatic compounds, demonstrates a unique ecological adaptation (Kim et al., 2010). These compounds, common environmental contaminants with potential human health risks, are efficiently degraded by mycobacteria. However, it’s intriguing that this associated gene family is notably absent in the majority of pathogenic bacteria. This raises questions about the ecological implications and potential genetic trade-offs associated with this adaptation in pathogenic versus non-pathogenic mycobacteria.

### 3.5 Variation in the presence and absence of virulence factors among *Mycobacterium* species

**Figure 4:**
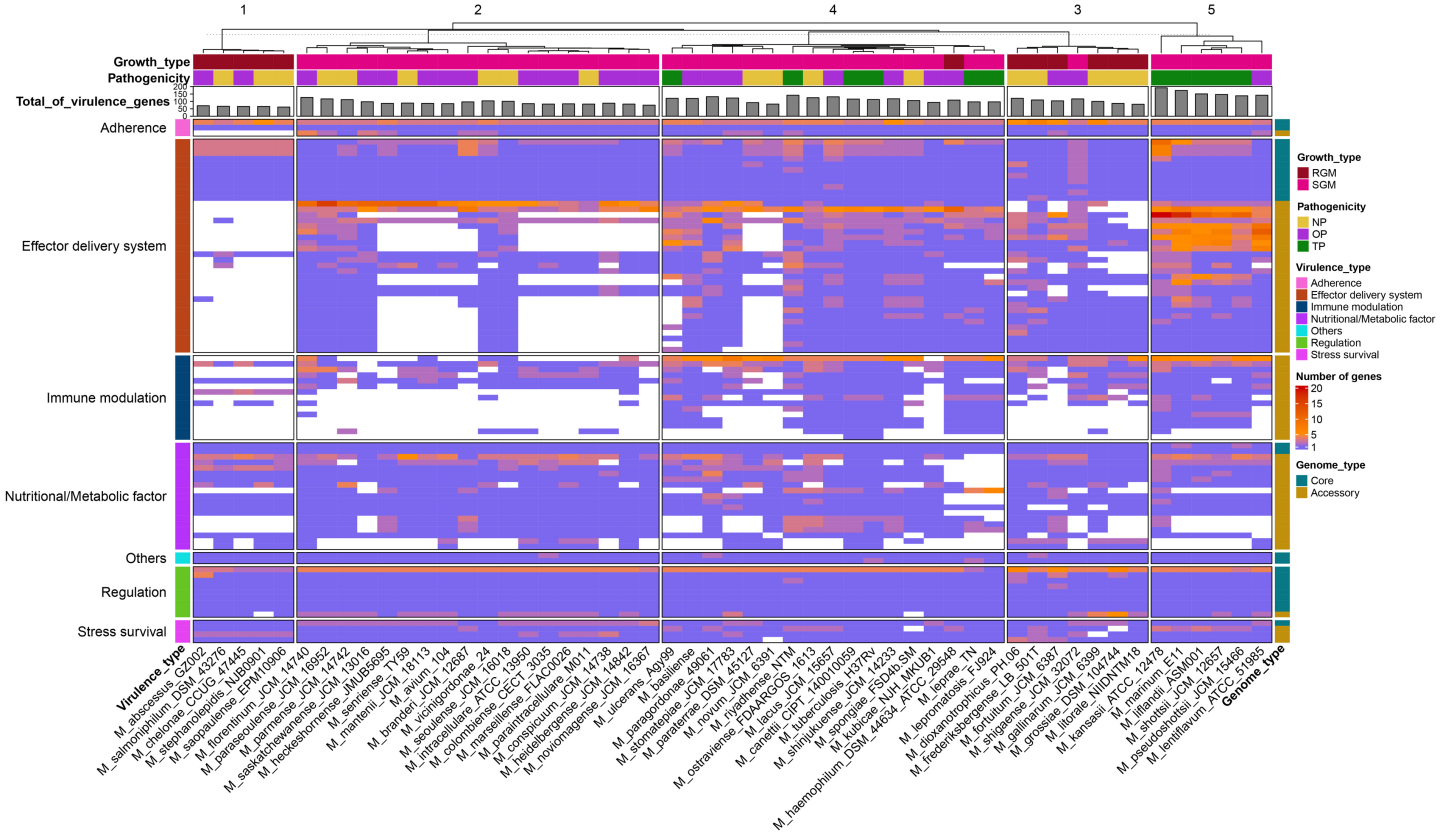
Presence-absence variation of virulence factors in each *Mycobacterium* species. The heatmap displays the number of virulence genes within corresponding gene families, represented by a color range, while white indicates the absence of the gene family. The virulence factors are color-coded based on VFDB classification on the left. Gene family types are indicated by color tiles on the right. Growth type and pathogenicity type annotations are provided as color tiles at the top of the heatmap. Additionally, the bar chart in the upper layer illustrates the total count of virulence genes for each *Mycobacterium* species.

The genomes of pathogens harbor specific virulence factors that bestow the organism with the capacity to manipulate host immune defenses, enhance its disease-causing potential, and profoundly influence the course of infections (Bo et al., 2018; Shariq et al., 2023). Consequently, gaining a comprehensive grasp of these virulence factors holds paramount importance for attaining valuable insights into the mechanisms that underlie the infection process. The utilization of the pan-genome approach provides a distinctive avenue to pinpoint diverse pathogenic virulence genes dispersed across various *Mycobacterium* species and to probe the distribution or absence of these virulence genes within the *Mycobacterium* genus. Within the scope of this study, a total of 90 virulence genes were identified across the 53 *Mycobacterium* species, encompassing 26 core genes and 64 accessory genes (Figure 4). The functionalities of these virulence genes were associated with effector delivery systems and adherence (38 genes), nutritional and metabolic factors (19 genes), immune modulation (15 genes), regulation (9 genes), stress survival (4 genes), and other functions (2 genes) (Supplementary Table S4).

The core virulence genes primarily serve regulatory roles (*phoP, whiB3, VF0257, mprB, ideR, relA, sigE, hspR*) and participate in effector delivery systems (*PE5, esxG, esxH, eccE3, mycP3, eccD3, eccB3, eccC3, espG3, eccA3, espR*). Cluster analysis unveiled that pathogenic bacteria (Figure 4, cluster 5) exhibited the highest prevalence of virulence genes, whereas species belonging to the *M. abscessus*-*M. chelonae* complex (cluster 1) had the lowest presence. Notably, substantial variations in the distribution of accessory genes associated with immune modulation and effector delivery systems were observed between cluster 5 and the other four clusters. Phthiocerol dimycocerosate, a compound produced through the collaboration of *ddrB, drrC, fadD22, lppx, mmpL7, ppsA, ppsE, Rv2949c, Rv2951c, Rv2954c, Rv2958c, Rv2959c* and *tesA*, serves as a vital constituent of the mycobacterial cell wall, aiding in the evasion of host immune detection and counterattack (Rens et al., 2021). Mycobacteria showcase a multitude of paralogous type VII secretion systems known as Esx-1 to Esx-5. Notably, Esx-1 exerts a pivotal function in facilitating the *in vivo* expansion of pathogenic mycobacteria, and Esx-3 is essential for mycobactin-mediated iron acquisition as well as for *in vitro* growth (Siegrist et al., 2009, 2014). The aforementioned findings underscore that the distinct distribution patterns of these virulence genes stand as pivotal genetic underpinnings contributing to the diversity in pathogenicity observed among various *Mycobacterium* species.

*M. tuberculosis* and *M. leprae* exhibit a range of common attributes while presenting minimal distinctions, with notable variations observed primarily in growth rate and disease phenotypes (Hussain, 2007). While nearly all virulence factors are present in both *M. tuberculosis* and *M. canettii*, several Nutritional/Metabolic factors are notably absent in the *M. leprae* and *M. lepromatosis* (Supplementary Table S4). Notably, the genes *panC* and *panD*, which are pivotal in pantothenate biosynthesis, demonstrate conservation across all mycobacterial species (Figure 4). Pantothenic acid, also referred to as vitamin B5, plays a fundamental role in the biosynthesis of crucial molecules such as coenzyme A and acyl carrier protein (ACP) (Sambandamurthy et al., 2002). On the other hand, *M. leprae* and *M. lepromatosis* lack twelve genes responsible for encoding mycobactin, including *mbtA, mbtB, mbtD, mbtE, mbtF, mbtG, mbtH, mbtI, mbtJ, mbtK, mbtM* and *mbtN*. Reddy et al. (2013) have reported that the disruption of mycobactin biosynthesis leads to the attenuation of both the growth and virulence of *M. tuberculosis*. Thus, the absence of these genes in *M leprae* and *M. lepromatosis* could contribute to the significantly sluggish growth rate exhibited by these organisms.

**Figure 5:**
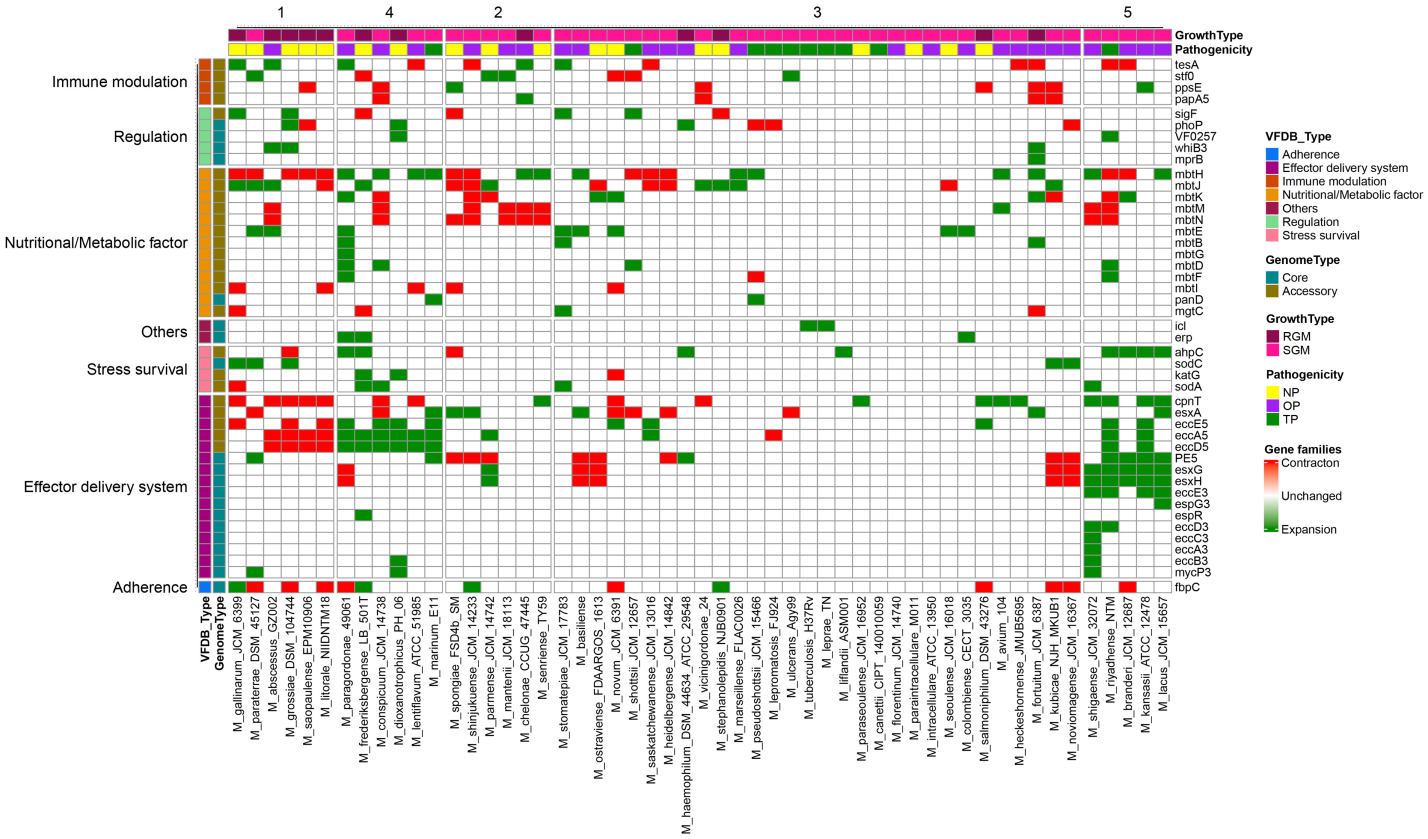
Variation in expansion and contraction of virulence gene families across *Mycobacterium* species. The heatmap illustrates the contraction (in red), expansion (in green), or unchanged status (in white) of virulence gene families for each mycobacterial species. The corresponding color tiles on the right denote the virulence and genome types of each gene family. Annotations for growth type and pathogenicity type are indicated at the top of the heatmap using color tiles.

There were 45 instances of expansion and contraction variation in virulence gene families across *Mycobacterium* species (Figure 5). Gene families associated with iron uptake factors and the Type VII secretion system exhibited reductions in rapid growth and non-pathogenic strains. However, these same gene families experienced substantial expansion in pathogenic or opportunistic pathogen strains. For instance, the gene families related to the ESAT-like secretion system 5 (ESX-5), including *EccA5, EccE5* and *EccD5*, expanded within cluster 4 (encompassing slow growth mycobacteria and pathogens), while undergoing contraction within cluster 1 (comprising rapid growth mycobacteria and non-pathogenic species). Deactivation of ESX-5 leads to a significant decrease in the secretion of PPE41, affecting the cell-to-cell migration of pathogenic mycobacteria (Abdallah et al., 2006). In *M. tuberculosis*, the EsxG-EsxH heterodimer has the ability to inhibit the host’s endosomal sorting complex required for transport (ESCRT) machinery, suggesting that *M. tuberculosis* might impede this specific host response mechanism (Mittal et al., 2018). Notably, these gene families underwent expansion in several opportunistic pathogens, including *M. shinjukuense*, *M. riyadhense*, *M. branderi*, *M. kansasii* and *M. lacus*. These findings indicate that the expansion and contraction of virulence genes during the evolutionary process are tightly interconnected with the pathogenicity of the *Mycobacterium* strains.

### 3.6 Presence or absence variation of antibiotics resistance genes for *Mycobacterium* species

It is widely recognized that infections caused by *M. tuberculosis* and non-tuberculosis mycobacteria (NTM) are often challenging to treat effectively due to their multidrug-resistant properties (Zumla et al., 2014; Ratnatunga et al., 2020). Bacteria can acquire resistance to antibiotics through genetic mutations or the acquisition of resistance genes from other organisms. Therefore, understanding the distribution of antibiotic resistance genes within bacterial populations is crucial for effective disease management and treatment. To investigate the distribution of antibiotics resistance genes of 53 *Mycobacterium* species, the proteome of each mycobacteria were analyzed based CARD database.

As depicted in Supplementary Figure S2, our analysis revealed the presence of eleven antibiotic resistance genes associated with resistance to ten distinct drug classes, including rifamycin, isoniazid-like antibiotics, glycopeptide antibiotics, and others. These resistance genes play a pivotal role in the ability of *Mycobacterium* species to withstand the effects of various antibiotics. These genes are linked to four primary mechanisms of drug resistance, encompassing antibiotic efflux, inactivation, antibiotic target alteration, and protection. Particularly noteworthy is the widespread distribution of antibiotic resistance genes related to rifamycin and isoniazid (*efpA, rpoB*), macrolide and penam (*mtrA*), and phosphonic acid (*murA*) across the 53 mycobacterial species. Intriguingly, a consistent distribution of antimicrobial genes was observed among almost all TP species, with the exception of *M. leprae* and *M. lepromatosis*, as well as RGM species. This suggests that many drug-resistant genes within the *Mycobacterium* genus are inherent to the strains and are potentially reinforced by the intrinsic multidrug resistance conferred by the presence of a lipid-rich outer membrane (Nasiri et al., 2017). However, it is worth noting that *M. tuberculosis* and NTM exhibit significant heterogeneity in drug resistance (Gopalaswamy et al., 2020), highlighting the necessity of tailoring antibiotic treatment regimens based on strain phenotypes in clinical settings. Furthermore, the presence of specific resistance genes in non-pathogenic species raises intriguing questions about the potential sources and mechanisms of resistance gene dissemination among *Mycobacterium* species.

This study provides a comprehensive analysis of the expansion and contraction patterns in gene families of different *Mycobacterium* species and their association with growth rate and pathogenicity. The identified gene family expansion/contraction events related to pathogenicity, such as the type VII secretion systems and mycobactin biosynthesis, have significant implications for a deeper understanding of the pathogenic mechanisms of *M. tuberculosis*. Previous comparative genomics studies of *M. tuberculosis* have primarily focused on single nucleotide variations, horizontal gene transfers, and sequence-level changes (Gautam et al., 2017; Coll et al., 2018). In the long course of evolution, it is noteworthy to investigate whether different *M. tuberculosis* lineages/sublineages have experienced gene family expansion and contraction events related to their adaptability. This study serves as a reference for gene family analyses in *M. tuberculosis* lineages and other species. However, this study has certain limitations. First, *Mycobacterium* encompasses at least 190 species, and a more extensive dataset of *Mycobacterium* species genomes may yield more precise results. Furthermore, the findings of this study need further experimental validation in the future.

## 4 Conclusion

In conclusion, our study delved into the intricate genomic dynamics underlying growth rate variations and pathogenicity shifts within the *Mycobacterium* genus. Through an extensive analysis of 53 species, we have unveiled the genetic determinants responsible for these crucial phenotypic traits. The pan-genome analysis revealed a dynamic nature of the *Mycobacterium* genus, with core and accessory genomes playing distinct roles in maintaining essential functions and facilitating adaptability, respectively. Notably, the core genome appeared to stabilize after a certain number of species, while the pan-genome continued to expand, underscoring the genus’ remarkable genetic diversity. Through the exploration of gene families associated with growth rate changes and pathogenicity shifts, we uncovered pivotal genetic determinants responsible for these phenotypic variations. Our findings underscore the significant role of gene family contractions, particularly those linked to nucleic acids, ions, and substance metabolism, in driving the intricate process of host adaptive evolution. Conversely, the expansion of virulence-associated gene families, notably encompassing the type VII secretion system and mycobactin biosynthesis, emerges as an equally pivotal determinant in shaping the pathogenicity landscape of mycobacteria. These dual mechanisms collectively highlight the dynamic interplay between genetic alterations and virulence factors that ultimately define the nuanced pathogenic potential of mycobacterial species. Moreover, our study delved into the intriguing landscape of antibiotic resistance genes, revealing their widespread presence across *Mycobacterium* species. The association of specific resistance genes with growth types and pathogenicity profiles highlighted their intrinsic nature in certain strains, potentially stemming from lipid-rich outer membranes.

Overall, this research provides valuable insights into the intricate interplay between genetic makeup, phenotypic traits, and pathogenicity in the *Mycobacterium* genus. These findings contribute to a deeper comprehension of the evolutionary dynamics that mold these bacterial populations. As we continue to decipher the complex interplay between genetics and phenotypic traits, we pave the way for potential advancements in diagnostics, therapies, and strategies for combating mycobacterial infections.

## Data availability statement

The original contributions presented in the study are included in the article/Supplementary material, further inquiries can be directed to the corresponding authors.

## Author contributions

XiaZ: Methodology, Writing – original draft, Investigation. QuL: Investigation, Writing – original draft, Data curation, Formal analysis. YL: Data curation, Investigation, Writing – review & editing. QiL: Data curation, Writing – review & editing. XinZ: Data curation, Writing – review & editing. XL: Writing – review & editing, Conceptualization. DC: Writing – review & editing, Conceptualization, Funding acquisition, Methodology, Supervision, Visualization, Writing – original draft.

## Footnotes

^1^ https://ftp.ncbi.nih.gov/genomes/
