# Supplementary material for: Contraction and expansion dynamics: deciphering genomic underpinnings of growth rate and pathogenicity in Mycobacterium
Source: Front Microbiol. 2023 Nov 23;14:1292897. doi: 10.3389/fmicb.2023.1292897 (PMC10701892; doi:10.3389/fmicb.2023.1292897)
Supplement: Supplementary file 2 [file Data_Sheet_1.docx]

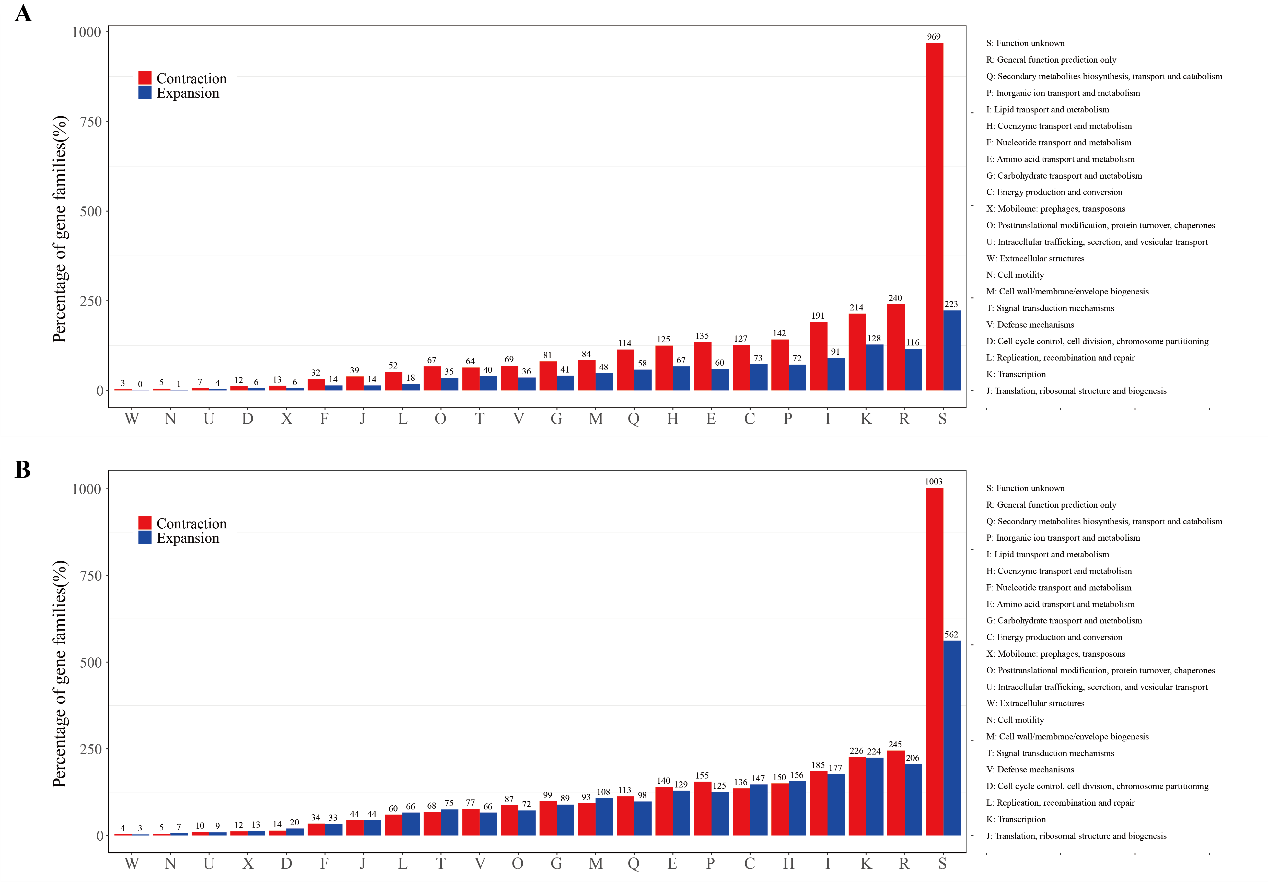


**Figure S1.** Distribution of COG categories for contraction and expansion gene families of inner nodes and lineage nodes.


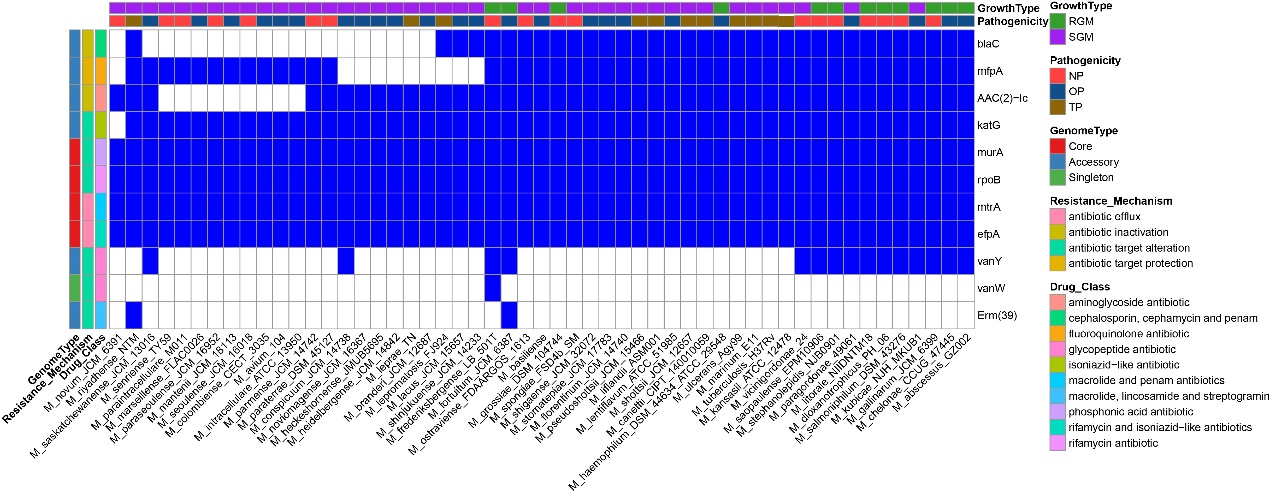


**Figure S2.** Presence/absence variation of antibiotics resistance genes in each *Mycobacterium* species. The heatmap illustrates the presence (blue) and absence (white) of individual antibiotic resistance genes. Additional information is displayed through color-coded tiles, indicating genome type, antibiotics resistance mechanism, and drug class on the left. Similarly, growth type and pathogenicity type are annotated with color tiles at the top.
